# Anterior internal versus external fixation of unstable pelvis fractures was not associated with discharge destination, critical care, length of stay, or hospital charges

**DOI:** 10.1007/s00590-024-03985-9

**Published:** 2024-05-21

**Authors:** Ian G. Hasegawa, Brandan Sakka, Andrew M. Duong, Li Ding, Monica D. Wong, Joshua L. Gary, Joseph T. Patterson

**Affiliations:** 1 Dpeartment of Orthopaedic Surgery, Queens Medical Center, Honolulu, Hawaii US; 2 Department of Orthopaedic Surgery, Keck School of Medicine of University of Southern California, 1520 San Pablo Street, Suite 2000, Los Angeles, CA 90033-5322 US; 3 Department of Population and Public Health Sciences, Keck School of Medicine of University of Southern California, Los Angeles, CA US; 4 Department of Surgery, Keck School of Medicine of University of Southern California, Los Angeles, CA US

**Keywords:** Pelvis fracture, Internal fixation, External fixation, Discharge, Home

## Abstract

**Purpose:**

Determine if anterior internal versus supra-acetabular external fixation of unstable pelvic fractures is associated with care needs or discharge.

**Methods:**

A retrospective cohort study was performed at two tertiary trauma referral centers. Adults with unstable pelvis fractures (AO/OTA 61B/61C) who received operative fixation of the anterior and posterior pelvic ring by two orthopedic trauma surgeons from October 2020 to November 2022 were included. The primary outcome was discharge destination. Secondary outcomes included intensive care unit (ICU) or ventilator days, length of stay, and hospital charges.

**Results:**

Eighty-three eligible patients were 38.6% female, with a mean age of 47.2 ± 20.3 years and BMI 28.1 ± 6.4 kg/m^2^. Fifty-nine patients (71.1%) received anterior pelvis internal fixation and 24 (28.9%) received external fixation. External fixation was associated with weight-bearing restrictions (91.7% versus 49.2%, p = 0.01). No differences in demographic, functional status, insurance type, fracture classification, or injury severity measures were observed by treatment. Internal versus external anterior pelvic fixation was not associated with discharge to home (49.2% versus 29.2%, p = 0.10), median ICU days (3.0 [interquartile range (IQR) 7.8 versus 5.5 [IQR 4.3], p = 0.14, ventilator days (0 [IQR 6.0] versus 0 [IQR 2.8], p = 0.51), length of stay (13.0 [IQR 13.0] versus 17.5 (IQR 20.5), p = 0.38), or total hospital charges (US dollars 180,311 [IQR 219,061.75] versus 243,622 [IQR 187,111], p = 0.14).

**Conclusions:**

Anterior internal versus supra-acetabular external fixation of unstable pelvis fractures was not significantly associated with discharge destination, critical care, hospital length of stay, or hospital charges. This sample may be underpowered to detect differences between groups.

**Level of Evidence:**

Therapeutic Level IV.

## Introduction

The incidence of traumatic pelvic ring injuries has increased over the last three decades [1–4]. Concurrently, greater awareness of these injuries, advances in resuscitation and surgical techniques, and shifts in surgeon preferences and training experiences have contributed to more patients surviving a pelvis fracture as well as more patients receiving surgical management for their fracture [2, 4–8]. As a consequence, the demand for post-acute care of pelvis fracture survivors has grown in magnitude and complexity [1, 9–11].

Discharge placement of patients admitted for acute pelvic fracture must consider preinjury function and living environment, support systems, financial resources and strain, insurance considerations, comorbid conditions and injuries, ongoing medical care needs, weight-bearing restrictions, and durable medical equipment requirements. Discharge to “home,” or previous living environment, after pelvic trauma has been associated with fewer readmissions, complications, and mortality, lower costs, and greater patient satisfaction [12, 13]. However, 40–60% of patients with pelvis fracture discharge to an acute inpatient rehabilitation (ARU) or skilled nursing facility (SNF) [1, 14]. Factors influencing discharge destination after acute pelvic fracture have not been well studied, particularly after surgical stabilization.

For unstable pelvis fractures, internal and external fixation methods exist for stabilizing the anterior pelvic ring, without clear consensus on the indications for either class of techniques [15–17]. Compared to external fixation, the purported benefits of internal fixation include decreased pain, increased stability of fractures and/or symphyseal disruptions, and improved mobilization [9–11, 18–21]. Surgeons also report imposing less restrictive weight-bearing precautions after anterior pelvis internal fixation [8]. Thus, anterior pelvis internal fixation versus external fixation may affect the probability that a patient discharges to home by affecting pain, ambulatory performance, evaluation by rehabilitation providers, and discharge recommendations.

The primary objective of this study was to determine if internal versus external fixation of the anterior pelvic ring, combined with posterior fixation, for adults with unstable pelvis fractures is associated probability of discharge to home. Secondary outcomes included use of critical care resources, length of stay, and hospital charges. We hypothesized that internal fixation is associated with greater likelihood of home discharge and decreased ICU days, ventilator days, length of stay, and hospital charges.

## Methods

A retrospective cohort of 83 patients aged ≥ 16 years admitted within 3 weeks of injury for unstable pelvic ring injury (AO/OTA 61B/61C) who received operative stabilization of both the anterior and posterior pelvic ring at one county Level 1 trauma center and one academic tertiary referral hospital by two fellowship-trained orthopedic traumatologists from October 2020 to November 2022 was identified from hospital case logs. Treatment was classified by supra-acetabular external fixation with 5 mm Schanz screws versus internal fixation by any combination of symphyseal plating and/or intramedullary screws. No patient was treated with an anterior subcutaneous internal fixator (In-Fix) or iliac crest external fixator placement. All patients received percutaneous 6.5 mm and/or 7.3 mm cannulated screw fixation for the posterior component of their pelvic ring injury. Postoperative weight bearing was at the discretion of the treating surgeon.

Eligibility, Young–Burgess fracture classification, and treatment classification were confirmed by review of preoperative and postoperative pelvis radiographs and computed tomography scans and operative by one orthopedic trauma fellow and adjudicated by the two attending surgeons. Three patients were excluded for inpatient status at the time of cohort selection. Clinical data were collected for 83 patients from an institutional trauma registry and manual chart review. Demographic variables, comorbidities, preinjury patient living environment, preinjury ambulatory status, Injury Severity Score (ISS), postoperative weight-bearing status (classified as full or weight bearing as tolerated, unilateral restrictions, or bilateral restrictions with or without weight bearing for transfers), and insurance type were collected.

The primary outcome was discharge to home (prior living environment) as described in discharge documentation. Secondary outcomes included ICU days and ventilator days as measures of critical care as well as hospital charges associated with the acute episode of care. Data analysis was performed by an independent biostatistician. Shapiro–Wilk tests were used to assess the normality of continuous data. Wilcoxon rank sum, Chi-square, and Fisher exact tests were used to assess differences in baseline covariates and outcomes between treatment groups. Multivariable regression analysis was used to determine associations between treatment and discharge destination, hospital length of stay, ICU days, ventilator days, and charges. Missing data were not imputed. Statistical significance was set at p < 0.05.

## Results

Fifty-nine (71.1%) patients received anterior pelvic ring internal fixation and 24 (28.9%) received supra-acetabular external fixation. No significant differences in age, sex, BMI, comorbid conditions, ambulatory status, preinjury living environment, insurance type, Young and Burgess fracture classification, ISS, or GCS were observed between treatment groups (Table 1). Patients treated with external fixation were more likely to have lower extremity weight-bearing restrictions compared to patients treated with internal fixation (91.7% vs 49.2%, p = 0.01). One in-hospital death occurred in the external fixation group secondary to multiorgan failure, pneumonia, and respiratory insufficiency.

**Table 1:** Demographic and comorbidity characteristics by anterior pelvis internal versus external fixation

	Overall n = 83		Anterior internal fixation n = 59	Anterior external fixation n = 24	p-value
Mean age (years)	47.2 ± 20.3		49.7 ± 20.8	41.0 ± 18.0	0.08
Sex (female)	32 (38.6%)		23 (39.0%)	9 (37.5%)	0.89
Mean BMI (kg/m^2^)	28.4 ± 6.4		28.1 ± 6.0	29.1 ± 7.5	0.70
Comorbid medical conditions					
Cardiovascular	14 (16.9%)		12 (20.3%)	2 (8.3%)	0.33
Diabetes	7 (8.4%)		5 (8.5%)	2 (8.3%)	0.99
Hepatic	1 (1.2%)		1 (1.7%)	0 (0.0%)	0.99
Neurologic	1 (1.2%)		1 (1.7%)	0 (0.0%)	0.99
Pulmonary	10 (12.0%)		10 (16.9%)	0 (0.0%)	0.06
Psychiatric	5 (6.0%)		4 (6.8%)	1 (4.2%)	0.99
Substance abuse	11 (13.3%)		7 (11.9%)	4 (16.7%)	0.72
Ambulatory pre-injury*	58 (100.0%)		40 (100.0%)	18 (100.0%)	-
Pre-injury living environment					0.12
Home	54 (87.1%)		36 (81.8%)	18 (100.0%)	
Sober living center	1 (1.6%)		1 (2.3%)	0 (0.0%)	
Homeless	7 (11.3%)		7 (15.9%)	0 (0.0%)	
Missing data	21		15	6	
Insurance					0.91
Medicaid	46 (63.0%)		32 (64.0%)	14 (60.9%)	
Private	24 (32.9%)		16 (32.0%)	8 (34.8%)	
Other	3 (4.1%)		2 (4.0%)	1 (4.3%)	
Missing data	10		9	1	
Mean GCS on arrival	13.0 ± 3.9	13.3 ± 3.8		12.4 ± 4.1	0.10
Mean ISS	25.1 ± 12.5	24.5 ± 12.0		27.1 ± 13.9	0.65
Pelvic ring injury classification					0.07
APC 2	19 (22.9%)	10 (16.9%)		9 (37.5%)	
APC 3	10 (12.0%)	6 (10.2%)		4 (16.7%)	
LC 1	18 (21.7%)	6 (10.2%)		4 (16.7%)	
LC 2	9 (10.8%)	9 (15.3%)		0 (0.0%)	
LC 3	3 (3.6%)	1 (1.7%)		2 (8.3%)	
Vertical shear	3 (3.6%)	3 (5.1%)		0 (0.0%)	
Combined	21 (25.3%)	16 (27.1%)		5 (20.8%)	
Post-op upper extremity weight bearing status					0.54
WBAT BUE	54 (65.1%)	38 (64.4%)		16 (66.7%)	
Unilateral	25 (30.1%)	19 (32.2%)		6 (25.9%)	
Bilateral	4 (4.8%)	2 (3.4%)		2 (8.3%)	
Post-op lower extremity weight bearing status					**0.01**
Bilateral weightbearing	51 (61.4%)	29 (49.2%)		22 (91.7%)	
Restriction unilateral restriction	26 (31.3%)	24 (40.7%)		2 (8.3%)	
WBAT BLE	6 (7.22%)	6 (10.2%)		0 (0.0%)	

*Data missing from 25 patients. BMI: body mass index. ORIF: open reduction internal fixation. APC: anterior–posterior compression type pelvic ring injury. GCS: Glasgow Coma Scale. ISS: injury Severity Scale. LC: lateral compression type pelvic ring injury. ORIF: open reduction internal fixation. WBAT: weight bearing as tolerated

On bivariable analysis, anterior pelvis internal versus external fixation was not significantly associated with discharge to home versus other location (49.2% vs. 29.2%, p = 0.096). Anterior pelvis internal versus external fixation was not significantly associated with any discharge destinations including home, ARU, SNF, or mortality (p = 0.16; Table 2).

**Table 2:** Clinical outcomes by anterior pelvis internal versus external fixation

	Overall n = 83	Anterior internal fixation n = 59	Anterior external fixation n = 24	*p* value
Median total ICU length of stay	4.0 (IQR 6.0)	3.0 (IQR 7.8)	5.5 (IQR 4.3)	0.14
Median total ventilator days	0 (IQR 6.0)	0 (IQR 6.0)	0 (IQR 2.8)	0.51
Median total hospital length of stay	14.0 (IQR 14.0)	13.0 (IQR 13.0)	17.5 (IQR 20.5)	0.38
Median total hospital charges (USD)	198,518 (IQR 223,255)	180,311 (IQR 219,061.75)	243,622 (IQR 187,111)	0.14
Discharge destination				0.16
Home	36 (43.4%)	29 (49.2%)	7 (29.2%)	
Inpatient rehabilitation	30 (36.1%)	20 (33.9%)	10 (41.7%)	
SNF	16 (19.3%)	10 (16.9%)	6 (25.0%)	
Inpatient mortality	1 (1.2%)	0 (0.0%)	1 (4.2%)	

ICU: intensive care unit. ORIF: open reduction internal fixation. USD: US dollar. SNF: skilled nursing facility.

On bivariable analysis, anterior pelvis internal versus external fixation was not significantly associated with median ICU days (3.0 [interquartile range (IQR) 7.8 versus 5.5 [IQR 4.3], p = 0.14; 6.0 [IQR 13.5] versus 6.0 [IQR 4.0], p = 0.73 among the 92 patients who admitted to an ICU) or days supported by a ventilator (0 [IQR 6.0] versus 0 [IQR 2.8], p = 0.51; 12 days (interquartile range (IQR) 10.5 days) vs. 6 days (IQR 3 days), p = 0.09) among the 43 patients who required mechanical ventilation, total hospital length of stay (13.0 [IQR 13.0] versus 17.5 (IQR 20.5), p = 0.38), or median hospital charges ($180,311 (IQR $219,061.75) vs $243,622 (IQR $187,111), p = 0.14). Bivariable regression analysis did not find that internal as compared to external fixation was significantly associated with hospital length of stay (incidence rate ratio [IRR] 0.81, 95% CI 0.56–1.18, p = 0.27) or ICU length of stay (IRR 0.99, 95% CI 0.56–1.75, p = 0.97). Similarly, internal fixation was not significantly associated with discharge to ARU (odds ratio [OR] 0.48, 95% confidence interval [CI] 0.16–1.48, p = 0.20) or SNF (OR 0.40, 95% CI 0.11–1.48, p = 0.17).

## Discussion

This retrospective investigation sought to compare the effect of internal versus external fixation of the anterior component of unstable pelvis fractures on discharge destination and resource utilization, including ICU days, ventilator days, and total charges for the index hospital admission. The cohort of 83 patients treated at two orthopedic trauma referral centers was similar between groups and all received posterior ring stabilization, strengthening the bivariable analyses. No advantage to internal fixation was observed for any of the outcomes at a statistical significance of α < 0.05. We observed a large, clinically relevant absolute risk difference of 20% in discharge to home which favored internal fixation of the anterior pelvic ring which did not reach statistical significance with this sample. However, we observed uneven confidence intervals about these estimates which suggest that with greater patient numbers this difference could be statistically and clinically significant. 

External fixation of the anterior pelvis offers a simple, fast, low-risk, and effective strategy for stabilizing the anterior component of unstable pelvis, providing adequate stability for transfers as well as pain relief [22–24]. Internal fixation of anterior pelvis fractures, by any combination of percutaneous antegrade anterior column screws, retrograde screws, open symphyseal plating, cerclage, or percutaneous symphyseal screws, imposes greater risk to neurovascular structures and requires additional operative and fluoroscopy time but also affords greater biomechanical stability [15, 18, 19]. Ostensibly, the greater stability provided by internal fixation decreases pain associated with mobilization. Conversely, while the bulk of external fixation interferes with perineal hygiene, donning of clothes, and ambulation [9–11, 18–21]. These characteristics of external fixation may interfere with mobilization, delay hospital discharge, and increase the risk of patients discharging to a destination other than home. In critically ill patients, anterior pelvic external fixation may also interfere with sitting the patient upright, with negative consequences for pulmonary toilet, risk of pneumonia, duration of ventilator and ICU requirements, and possibly mortality.

Previous studies have reported conflicting advantages to internal versus external fixation. Wardle et al. performed a systematic review of studies published between 1989 and 2015 and determined that anterior supplemental internal fixation of unstable pelvis fractures was associated with a lower incidence of fracture displacement > 1 cm and unsatisfactory outcomes, with similar rates of loss of any reduction, nonunion, and malunion [18]. Conversely, Baron et al. described no difference in Majeed scores of 70 (95% CI 28–100) with external fixation versus 79 (95% CI 36–100) with internal fixation (p = 0.28) in a single-center series of 58 patients, concluding the absence of a difference in long-term outcome supports the use of external fixation [19]. Buller et al. found a lower odds of discharge to an ARU or SNF facility associated with internal fixation in an analysis of the Nationwide Inpatient Sample of hospital discharges [1]. The authors also observed that both internal and external fixation of unstable pelvic ring injuries were associated with non-routine hospital discharge, which likely reflects the severity of pelvic fractures treated with anterior stabilization. However, the validity of this database investigation cannot be confirmed without radiographic evidence of appropriate fracture and treatment classification.

In the present study, the 55.4% rate of non-home discharge after the surgical fixation of an unstable pelvic ring injury is comparable to the relatively high rates previously reported and highlights the need for strategies to increase discharges to home. Buller et al. noted a 40% rate of non-routine discharge to a short- or long-term care facility in their nationwide analysis of 1,464,458 pelvic ring injuries, 9% of which were treated with internal or external fixation [1]. Discharge to a care facility exceeded 60% in a cohort of 572 pelvic ring and acetabular fractures treated with open reduction internal fixation [25]. Similarly, discharge to a SNF facility ranged from 62–65% in a separate nationwide analysis of 123,936 low-energy pelvic ring injuries treated non-operatively [5]. Non-home discharges after pelvis fracture have been associated with older age, female gender, preoperative bleeding disorder, transfer from outside emergency department, and American Society of Anesthesiologists physical status classification score > 2 [1].

This study has limitations common to retrospective investigations at a single center, including selection bias, missing data, a limited sample size, and ultimately a lack of statistical power. Bivariate regression analyses suggest that internal fixation may have substantial effects on probability of home discharge as well as reduced ICU days and length of stay, but confidence in these estimates is poor due to the small sample. Of note, a minimal clinically important difference for discharge to home after pelvis fracture is not currently defined. Classification bias may result from combining multiple internal fixation strategies into a single designation, which did not account for variability in efficacy and complications within the internal fixation group. We did not examine differences in fracture characteristics between groups, which may have influenced the treatment decision and confounded the relationship between treatment and outcomes. Conversely, the validity of the present study is strengthened by the radiographic classification of injuries and treatments by experienced pelvis fracture surgeons and the use of prospectively collected registry data to characterize patients, interventions, and outcomes, while the conduct at two different centers affords some generalizability.

In conclusion, unstable pelvic ring disruptions treated with anterior and posterior pelvic ring fixation are associated with a high rate of discharge to post-acute rehabilitation or care facilities. Internal fixation of the anterior pelvic ring disruption was not associated with significant advantages over external fixation with regard to discharge home, critical care resource utilization, length of stay, or hospital charges. However, these observations may be underpowered to identify a clinically relevant difference. A prospective, multicenter evaluation of the treatment effect of internal versus external fixation of unstable pelvic fractures may be indicated.
